# IL-33/ST2 Signaling and Microglial Functional-State Transitions After Spinal Cord Injury: Direct Evidence and Mechanistic Hypotheses

**DOI:** 10.3390/ijms27188254

**Published:** 2026-09-16

**Authors:** Ziyu Ma, Zicheng Lu, Zipeng Zhou, Tianhao Wang, Jinhui Zhang, Yifei Ma, Ruihan Niu, Licheng Zhang, Junhao Deng, Yongfei Zhao

**Affiliations:** Senior Department of Orthopedics, Chinese PLA Hospital, Beijing 100853, China; mzy301spine@163.com (Z.M.); lzc20210101@126.com (Z.L.); zhouzipeng1990@163.com (Z.Z.); wangtianhao@301hospital.com.cn (T.W.); zhangjinhui301@hotmail.com (J.Z.); myfstayoff@126.com (Y.M.); 15230820949@163.com (R.N.); zhanglcheng301@163.com (L.Z.)

**Keywords:** spinal cord injury, tissue repair, IL-33, microglial heterogeneity

## Abstract

Spinal cord injury (SCI) triggers a complex and evolving cascade of pathological events in which neuroinflammation and microenvironmental imbalance critically constrain repair. Among the cellular mediators, microglia exhibit remarkable functional plasticity, transitioning across diverse states that can either support tissue repair or exacerbate secondary damage. However, the mechanisms governing this dynamic reprogramming remain incompletely understood, limiting the development of effective immunomodulatory strategies. Recent evidence identifies the interleukin-33 (IL-33)/ST2 axis as an emerging, context-dependent regulator linking immune responses to neural repair. Rapidly released as an alarmin following injury, IL-33 modulates microglial function across spatiotemporal dimensions. Here, we propose a two-stage working model integrating direct evidence from SCI with extrapolated insights from broader central nervous system (CNS) pathologies. While acute IL-33 signaling is directly demonstrated in SCI models to limit early neuroinflammation and contain tissue damage, its putative role in driving a subsequent reparative microglial phenotype—encompassing metabolic adaptation and specialized phagocytic clearance—remains a hypothesis largely inferred from brain injury and neurodegenerative paradigms. Furthermore, we explore how IL-33 might modulate intercellular crosstalk with regulatory T cells and astrocytes, emphasizing that these downstream reparative mechanisms require definitive validation within the specific microenvironment of the injured spinal cord. In this Review, we synthesize current advances in the understanding of SCI pathology, microglial heterogeneity, and IL-33-mediated signaling. We highlight how IL-33 integrates inflammatory, metabolic, and transcriptional programs to drive microglial functional reprogramming, and we evaluate emerging therapeutic strategies targeting this pathway. Despite promising preclinical findings, challenges remain in optimizing delivery, timing, and safety. A deeper understanding of the IL-33/ST2 axis and its context-dependent effects may enable precise immunomodulation, offering new avenues to overcome barriers to regeneration and improve functional recovery after SCI.

## 1. Introduction

Spinal cord injury (SCI) is a devastating disorder of the central nervous system. Globally, SCI accounts for approximately 0.9 million new cases annually, with an overall age-standardized incidence rate of 11.5 per 100,000 population. Men are disproportionately affected compared to women, exhibiting both higher absolute incidence (~520,000 vs. ~380,000 annual cases) and higher age-standardized incidence rates (~14.8 vs. ~8.3 per 100,000 population). SCI results in permanent motor, sensory, and autonomic deficits, imposing lifelong medical and psychosocial burdens on patients [1]. Despite major advances in acute care and complication management, meaningful neurological recovery remains elusive. Therapeutic intervention following traumatic SCI is substantially hindered by a series of complex pathophysiological hurdles, including the structural disruption and subsequent dysfunction of the blood–spinal cord barrier (BSCB), the dense physical deposition of astrocytic and fibrotic scar tissue, and an inhibitory immune microenvironment [2]. Overcoming these barriers requires both a deeper understanding of the molecular basis of injury and repair and precise targeting of key cellular and molecular regulators.

Microglial responses after SCI exhibit remarkable heterogeneity shaped by transcriptional states, spatial distribution, and localized microenvironmental signals. Rather than following a strictly temporal or binary dichotomy, microglial subsets at the lesion core primarily engage in phagocytic clearance of myelin debris and damaged cell remnants, contributing to scar border formation and tissue containment. Conversely, microglial subpopulations in the peri-lesion and spared spinal tissue display distinct activation profiles, where persistent secretion of pro-inflammatory mediators and impaired metabolic homeostasis can exacerbate neurotoxicity. Understanding these specialized microglial states—defined by their spatial niches and distinct functional roles—is crucial for developing targeted neuroprotective interventions [3]. Emerging evidence indicates that microglia do not conform to a simple pro-inflammatory versus reparative dichotomy. Instead, they exhibit pronounced heterogeneity, with functional states that dynamically evolve across spatial and temporal dimensions after injury. The mechanisms governing these transitions remain poorly defined. A central challenge in the field is therefore to direct microglial responses toward phenotypes that support repair.

In this context, Interleukin-33 (IL-33), a pleiotropic alarmin, has emerged as a key regulator. Within the central nervous system, IL-33 is constitutively stored in the nuclei of mature oligodendrocytes and astrocytes [4,5], with endothelial cells also contributing upon neurovascular disruption [6]. Its release and subsequent functions are tightly coupled to temporal injury phases. During the acute phase following mechanical trauma, necrotic cell death triggers the rapid, passive release of pre-stored IL-33 within 24 h, which limits inflammatory spread [7]. However, chronic neuroinflammation can drive sustained de novo IL-33 expression, contributing to maladaptive fibrotic remodeling and persistent glial reactivity [6]. Upon binding to its receptor ST2 (IL-1RL1), IL-33 activates pro-survival signaling pathways in neurons, including PI3K/AKT, and reprograms microglial function toward an anti-inflammatory and reparative phenotype. In addition, accumulating evidence from CNS injury models suggests that IL-33 may enhance microglial phagocytic activity, potentially facilitating the clearance of inhibitory myelin debris [8]. This spatiotemporal duality highlights IL-33 as both a key mediator linking immune responses to neural repair and a promising therapeutic target.

Here, we systematically integrate current knowledge on SCI pathology, microglial heterogeneity, and the multifaceted roles of IL-33. We further examine the molecular basis of their interactions and discuss their translational potential, with the aim of providing new insights into overcoming the therapeutic barriers to spinal cord repair.

### Literature Search Strategy and Narrative Synthesis Framework

While a systematic search strategy was employed to ensure literature rigor and reproducibility, this article is fundamentally structured as a narrative review. This format was deliberately chosen to accommodate our tiered evidence framework, allowing for the critical integration of direct traumatic SCI data (Tier 1) with extrapolated mechanistic hypotheses derived from related CNS pathologies (Tiers 2 and 3). Consequently, the following selection criteria were applied to construct a comprehensive narrative synthesis rather than a formal systematic review.

To ensure an objective and comprehensive synthesis, the literature published between January 2010 and May 2026 was retrieved from PubMed, Web of Science, and Embase. Search terms combined Medical Subject Headings (MeSH) and free text: (“spinal cord injury” OR “SCI”) AND (“microglia” OR “microglial heterogeneity” OR “functional states”) AND (“interleukin-33” OR “IL-33” OR “ST2”).

Studies were included if they met the following predefined criteria: (1) study types and species: in vivo studies utilizing mammalian models (e.g., mice, rats, non-human primates) or human clinical/post-mortem tissues, alongside relevant in vitro mechanistic assays; (2) SCI models: standardized traumatic paradigms, including contusion, compression, and transection; (3) outcome measures: quantified assessments of microglial transcriptional/metabolic states, phagocytic dynamics, or IL-33/ST2-dependent tissue remodeling and functional recovery.

To ensure reproducibility, “rigorous microglial characterization” was strictly operationalized. Included studies were required to definitively distinguish resident microglia from infiltrating monocyte-derived macrophages. Methodologically, this required the use of single-cell RNA sequencing, genetic fate-mapping (e.g., *Cx3cr1-CreER* lineage tracing), multi-parameter flow cytometry, or high-resolution multiplexed immunohistochemistry on preserved spinal cord sections. Crucially, histological and cytometric validations had to incorporate resident microglia-specific markers (e.g., *TMEM119*, *P2RY12*, *Hexb*, or *Sall1*) rather than relying solely on pan-macrophage/myeloid markers (e.g., *Iba1*, *CD68*, *CD11b*), which cannot differentiate cellular ontogeny in the injured parenchyma.

**Figure 1:**
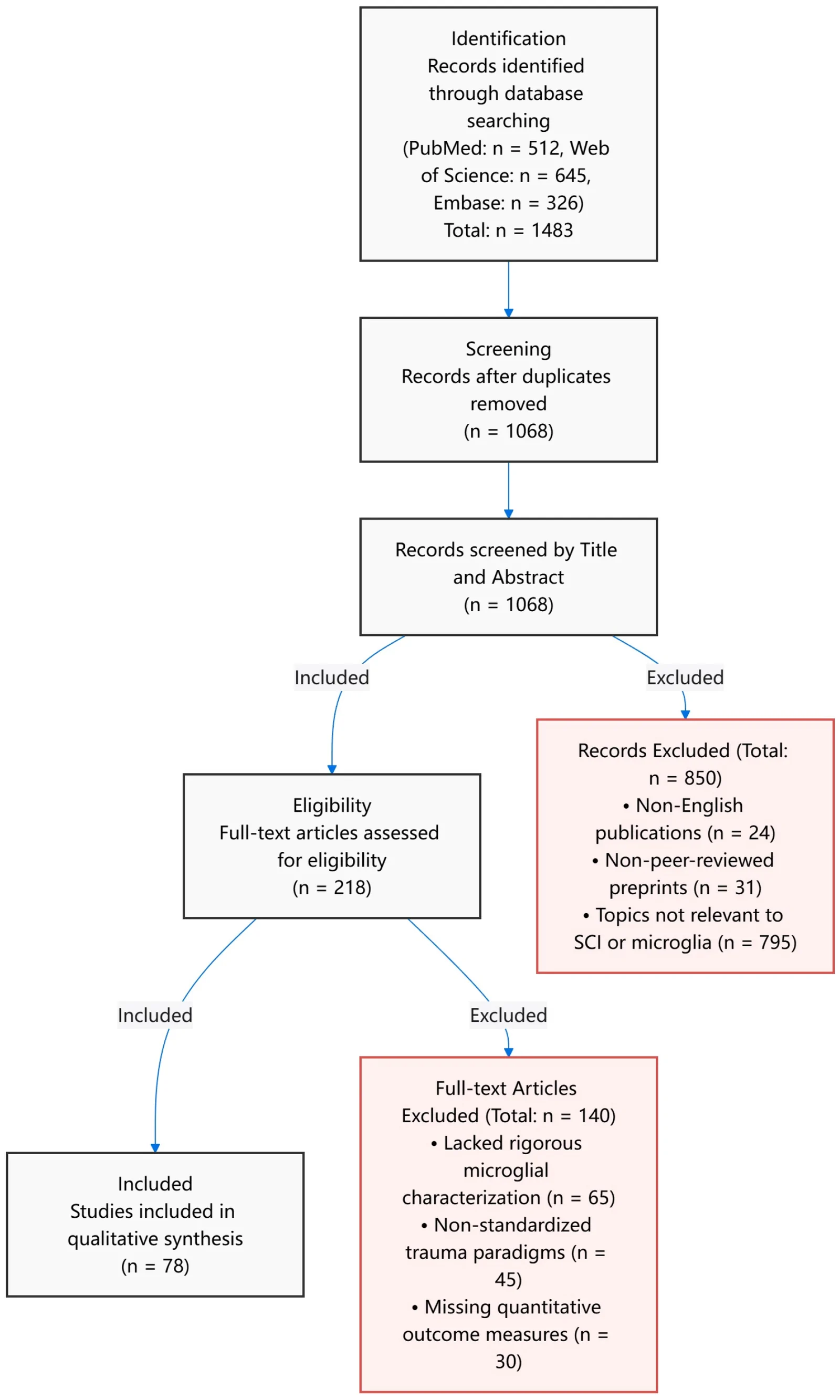
Flowchart of literature search and selection strategy for narrative synthesis.

Exclusion criteria comprised: non-peer-reviewed preprints, non-English publications, and studies lacking the aforementioned rigorous cell-type resolution. A detailed summary of the literature screening process is provided in Figure 1.

Flow diagram detailing the identification, screening, and inclusion of relevant studies published between January 2010 and May 2026 used to construct this narrative review. An initial search across PubMed, Web of Science, and Embase yielded 1483 records. After removing duplicates, 1068 records were screened by title and abstract, leading to the exclusion of 850 records (non-English publications, non-peer-reviewed preprints, or topics irrelevant to SCI or microglia). Full-text assessment of the remaining 218 articles resulted in the exclusion of 140 studies due to a lack of rigorous microglial characterization (e.g., distinguishing resident microglia from monocyte-derived macrophages), use of non-standardized trauma paradigms, or missing quantitative outcome measures. Ultimately, 78 studies met all predefined eligibility criteria and were included in the final qualitative synthesis to evaluate direct SCI evidence and extrapolated insights from related CNS pathologies.

## 2. Pathophysiology and Immune Microenvironment of SCI

### 2.1. Phases of Injury and Pathological Features

To systematically evaluate the context-dependent functions of microglial subpopulations and IL-33 signaling established above, it is essential to first contextualize the temporal landscape of SCI pathobiology. Traumatic SCI initiates a complex pathophysiological cascade traditionally operationalized into acute, subacute, and chronic phases. Because temporal definitions vary significantly between preclinical rodent models and clinical consensus guidelines, we establish a standardized murine timeline for this Review to align with the primary transcriptomic evidence discussed herein (summarized in Table 1). Specifically, we define the acute phase as 0 to 3 days post-injury, the subacute phase as 3 to 28 days (4 weeks), and the chronic phase as beyond 28 days. Crucially, these phases do not represent isolated, mutually exclusive windows; instead, they operate along a dynamic biological continuum with extensive temporal overlap.

Notably, neuroinflammation is not confined to the subacute phase. Rather, it is rapidly initiated within minutes of mechanical trauma during the acute phase (concurrent with hemorrhage, ischemia, and oxidative stress), reaches peak cellular reactivity and phenotypic divergence during the subacute window, and persists as sustained, low-grade chronic neuroinflammation alongside astroglial/fibrotic scarring and axonal regeneration failure. Recognizing this fluid temporal continuum is essential when evaluating microglial functional evolution across injury stages [9]. Mechanical trauma initially causes direct tissue damage, including neuronal compression, vascular disruption, axonal transection, and membrane rupture. This primary insult initiates a cascade of secondary events. Within the acute phase (0–3 days), profound alterations occur: neuronal loss and transcriptional suppression are prominent, while astrocytes, microglia, and oligodendrocyte precursor cells (OPCs) become activated and proliferative. Concurrently, vascular remodeling is dynamic, and inflammatory cells—particularly neutrophils—rapidly infiltrate the lesion, peaking within 1–3 days [10].

Within minutes of injury, microhemorrhages arise in the central gray matter and spread radially and longitudinally over subsequent hours [11]. This leads to marked swelling at the lesion site, often occupying the full diameter of the spinal canal. When tissue pressure exceeds venous pressure, autoregulation fails, resulting in neurogenic shock, systemic hypotension, and exacerbated ischemia [12]. Disruption of the blood–central nervous system barrier further facilitates immune cell infiltration. Meanwhile, antioxidant defenses—including superoxide dismutase (SOD), catalase, and glutathione peroxidase—are insufficient to counteract excessive reactive oxygen species (ROS), leading to membrane and organelle damage [13]. These events collectively activate resident glial cells and infiltrating leukocytes, amplifying inflammatory signaling and aggravating acute injury [3].

During the subacute phase (3–28 days), partial neuronal recovery is observed, while astrocytes transition into reactive states characterized by expression of Vimentin, GFAP, and Nestin, peaking at approximately day 3. Microglia exhibit a second activation wave around day 14, coinciding with a secondary decline in neuronal and astrocyte populations. CD3^+^ lymphocytes emerge after day 3, and endothelial cells continue to remodel [14]. Inflammation during this stage is a major driver of secondary injury. Activated microglia and astrocytes contribute to tissue remodeling, scar formation, and cavity development. The resulting scar, together with inhibitory molecules in the lesion microenvironment, forms a structural and molecular barrier to axonal regeneration and circuit reconstruction [15,16].

The chronic phase (>28 days) is characterized by sustained neuronal and astrocytic loss, persistent microglial activation with disease-associated transcriptional signatures (including genes linked to Alzheimer’s disease), ongoing leukocyte infiltration, and chronic inflammation. Astrocyte-derived scar tissue also serves to spatially confine inflammatory spread beyond the lesion core [17].

### 2.2. Immune Microenvironment After SCI

The immune microenvironment consists of immune cells, inflammatory mediators, and chemokines. Following SCI, a sustained immune–inflammatory response develops at the lesion site. This response involves microglia, macrophages, neutrophils, lymphocytes, and astrocytes, together with their secreted factors, intercellular interactions, inflammatory signaling, and extracellular matrix remodeling. Collectively, these processes disrupt microenvironmental homeostasis within the injured spinal cord.

Among these components, microglia represent one of the most dynamically responsive cell populations. They are rapidly activated after injury, produce a broad spectrum of pro-inflammatory mediators, and exhibit two major waves of activation, peaking at days 3 and 14 post-injury. The first wave is primarily associated with acute inflammation, whereas the second likely reflects secondary injury and persistent inflammatory signaling [18]. Notably, activated microglia display diverse transcriptional and functional states.

High-resolution single-cell RNA sequencing (scRNA-seq) performed by Brennan et al. [19] revealed that microglial activation following SCI is not a uniform process, but instead involves time-dependent transcriptional reprogramming into 11 distinct functional subpopulations. These defined subsets include homeostatic microglia expressing *P2ry12* and *Csf1r*, proliferative subpopulations enriched in ribosomal gene expression, non-lipid phagocytic subsets marked by *Lgals1*, and lipid-processing subsets characterized by *Lpl* and *Ctsd* expression. Each subpopulation dynamically shifts over time to govern specialized biological processes, including proliferation, debris phagocytosis, lipid and iron metabolism, antigen processing, and localized immune regulation [19,20]. Under baseline conditions, homeostatic microglia predominate. By 7 days post-injury, they are largely replaced by proliferative, non-lipid phagocytic, and lipid-processing subsets, reflecting a functional shift toward rapid expansion, efficient debris clearance, and early lipid metabolism. By 28 days, lipid-processing microglia become dominant, accompanied by increased surveillance and iron-handling subsets, while non-lipid phagocytic populations nearly disappear. This transition reflects a shift toward sustained lipid metabolism, iron homeostasis, and tissue surveillance during chronic repair.

Beyond resident microglia, peripheral myeloid populations—predominantly monocyte-derived macrophages (MDMs)—exhibit defined spatiotemporal recruitment patterns across the compromised blood–spinal cord barrier (BSCB). Within 1–3 h after injury, circulating monocytes are rapidly recruited to the lesion site. Between 6 and 12 h, these infiltrating monocytes expand and differentiate into primary tissue macrophages, accompanied by a local surge in cytokines such as TNF-α, IL-1β, and IL-6. Concurrently, resident and recruited dendritic cells initiate antigen presentation.

Crucially, high-resolution single-cell profiling has rendered the traditional linear M1-to-M2 polarization paradigm obsolete in the context of neurotrauma. From 24 h to 7 days post-injury and beyond, these myeloid cells do not undergo a simple binary phenotypic shift. Instead, they execute coexisting and dynamically changing inflammatory, phagocytic, lipid-handling, and repair-associated programs. Furthermore, it is imperative to distinguish the responses of infiltrating MDMs from those of resident microglia. While both lineages converge on shared transcriptional modules essential for myelin debris clearance (such as *Trem2* and *Apoe*), they preserve distinct ontogeny-driven trajectories and spatial niches. Infiltrating MDMs are predominantly confined to the highly necrotic lesion core, where they sustain robust antigen-presenting and effector-inflammatory signatures. Conversely, resident microglia primarily populate the peri-lesion borders to orchestrate physical scar containment, subsequently evolving into specialized lipid-processing and senescent subsets during chronic stages [14,19,21,22].

In contrast to the rapid innate immune response, components of adaptive immunity engage in a delayed, highly regulated manner. B and T lymphocytes begin infiltrating the injured spinal cord parenchyma approximately 3 days post-injury, driven by earlier dendritic cell antigen presentation. Over subsequent days to weeks, naive T cells undergo lineage-specific differentiation into Th1, Th2, Th17, and regulatory T (Treg) subsets, exerting divergent pro- or anti-inflammatory effects on the microenvironment. Concurrently, B cells contribute to sustained local immune regulation and potential secondary damage through antibody production and cytokine secretion [14,21,23].

Although multiple immune cell types display dynamic changes after SCI, microglial functional remodeling remains the most prominent feature of the evolving immune microenvironment.

### 2.3. Limitations in Current Repair Strategies

Current approaches to SCI repair include tissue engineering, pharmacological interventions, surgery, combination therapies, and emerging clinical trials. Tissue engineering strategies employ neurotrophic factors such as nerve growth factor (NGF) and brain-derived neurotrophic factor (BDNF), stem cell transplantation (including embryonic stem cells (ESCs), induced pluripotent stem cells (iPSCs), and mesenchymal stem cells (MSCs)), and biomaterial scaffolds such as 3D-printed or decellularized matrices to support regeneration.

Pharmacological agents, most notably high-dose methylprednisolone sodium succinate (MPSS), were historically administered with the rationale of dampening acute neuroinflammation and secondary cellular injury. However, the clinical efficacy and safety of MPSS remain highly contentious. While post-hoc analyses of landmark trials (such as NASCIS II and III) suggested modest neurological gains only when initiated within a narrow therapeutic window (<8 h post-injury), extensive subsequent evidence has raised major safety concerns [24]. Consequently, MPSS is currently regarded as a weak treatment option requiring rigorous risk-benefit assessment rather than a definitive therapy, underscoring the pressing clinical need for safer, more targeted neuroprotective modalities. Surgical strategies primarily focus on early decompression to stabilize the spine and relieve pressure. Combination therapies aim to enhance efficacy by integrating multiple modalities. Early clinical studies, including those using human embryonic stem cells (hESCs) and autologous MSCs, have demonstrated preliminary safety and functional benefits [25,26].

However, important limitations remain. Age is frequently overlooked, as most preclinical studies rely on young animals despite the reduced regenerative capacity associated with aging [27]. The role of adaptive immunity, particularly B and T cells, in SCI repair remains controversial, and the interactions among immune, glial, and vascular cells are incompletely understood [22]. In addition, crosstalk between myeloid cells (microglia and macrophages) and lymphocytes, such as CD8^+^ T cells, appears critical but mechanistically unresolved [19].

Finally, although broad immunomodulatory strategies demonstrate robust neuroprotective efficacy in preclinical settings, their successful clinical translation remains severely hampered. It is fundamentally flawed to infer direct clinical applicability solely from standardized experimental models. These highly controlled, sterile murine paradigms often fail to recapitulate the profound biological heterogeneity, variable injury biomechanics, and systemic immunological complexities inherent to human SCI. This translational disconnect underscores the urgent need for highly targeted, microenvironment-specific interventions rather than generalized immune suppression or activation.

## 3. Dynamic Regulation of Microglia in SCI

### 3.1. Microglial Subpopulations and Functional Diversity

The inflammatory response to SCI involves a complex interplay of immune cells, among which microglia act as the central regulators. Following injury, microglia rapidly activate and diversify into multiple functional subpopulations that exert distinct roles in inflammation and tissue repair [19,23]. Several distinct microglial subsets have been characterized across various CNS pathologies, though their direct translation to traumatic SCI remains variable. For instance, CD11c^+^ microglia, which emerge during late embryogenesis and require osteopontin (OPN) for phenotypic stability, exhibit robust phagocytic and proliferative capacities in neurodevelopmental and Alzheimer’s disease (AD) models [28]. However, their definitive validation as a distinct, early-responder subpopulation in the context of primary SCI is currently lacking. Similarly, dark microglia, characterized by ultrastructural stress features, are enriched in AD and chronic stress models, yet their specific presence and functional relevance within the injured spinal cord remain elusive [29,30]. Furthermore, repair-associated microglia (RAM) have been shown to support blood–brain barrier integrity and promote angiogenesis via VEGF-A secretion; however, this mechanism is derived exclusively from indirect evidence in cerebrovascular injury paradigms [31]. While it is highly plausible that analogous microglial subsets operate to stabilize the blood–spinal cord barrier (BSCB) during early SCI recovery, this extrapolation demands rigorous, spinal cord-specific empirical confirmation.

Microglial activation following central nervous system (CNS) pathobiology involves sophisticated state transitions. The canonical ‘disease-associated microglia’ (DAM) phenotype—originally defined in Alzheimer’s disease (AD) models—is characterized by the downregulation of homeostatic markers (e.g., *P2ry12*, *Cx3cr1*) and sequential activation of genes governing lipid metabolism and phagocytosis (e.g., *Trem2*, *Apoe*, *Lpl*, *Ctsd*). In the context of traumatic SCI, while classic amyloid-clearing mechanisms are not relevant, microglial subpopulations acquire DAM-like transcriptional signatures [32]. These DAM-like cells in SCI respond to massive tissue destruction by orchestrating myelin debris engulfment, lipid degradation, and localized tissue containment. Crucially, given etiology- and niche-specific divergence, microglial states in SCI represent distinct injury-induced transcriptional programs rather than an exact equivalence to AD-defined DAMs. A detailed summary of microglial subpopulations, their defining markers, model origins, and direct validation status in SCI is provided in Table 2.

Other subsets, such as plaque-associated microglia (PAM), appear to be disease-specific to AD, whereas white matter-associated microglia (WAMs) are highly relevant to SCI due to their role in maintaining myelin and oligodendrocyte homeostasis [33,34]. Together, these findings underscore the functional heterogeneity of microglia and their context-dependent specialization in SCI.

### 3.2. Physiological and Pathological Roles of Microglia

Under physiological conditions, microglia maintain CNS homeostasis through immune surveillance, trophic support, and synaptic remodeling. They interact closely with neurons and glial cells, respond to environmental cues, and regulate neural circuit integrity [35]. After SCI, microglia rapidly adopt phagocytic and inflammatory phenotypes. In the acute phase, they efficiently clear myelin debris via TREM2/DAP12 signaling and recognition of phosphatidylserine on apoptotic cells, thereby limiting tissue damage [36,37,38].

**Figure 2:**
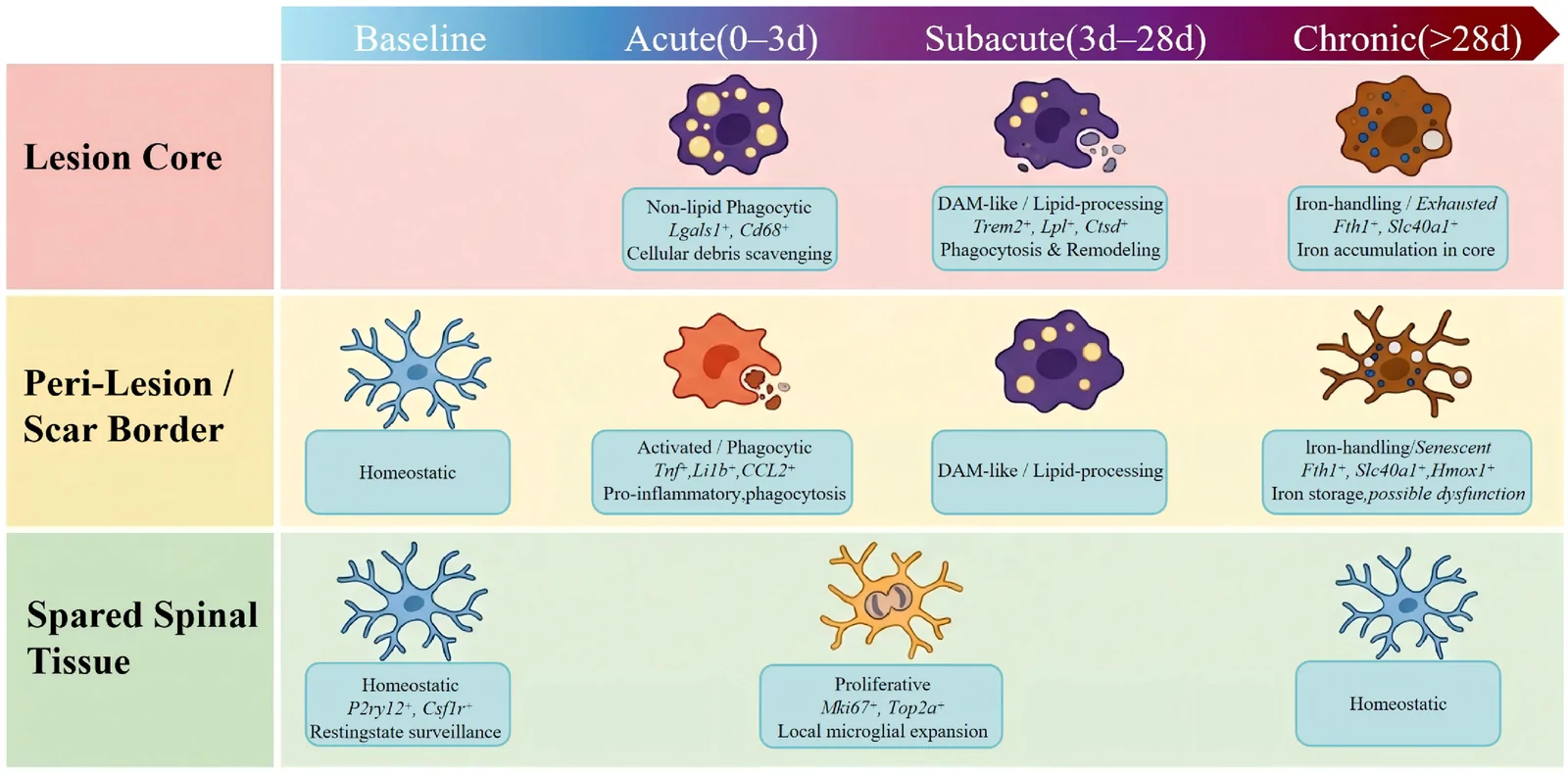
Spatiotemporal state transitions and functional heterogeneity of microglia following SCI.

Beyond acute phagocytosis, aberrant microglial synaptic pruning has emerged as a potential driver of secondary damage. While complement-mediated synaptic elimination was originally defined in neurodegenerative diseases [39] and neuropathic pain [40], recent direct evidence in SCI confirms that lipid-accumulating microglia utilize C1q-dependent mechanisms to inappropriately strip functional synapses, thereby impairing motor recovery [41] (Figure 2).

During the chronic phase of SCI, the microglial response evolves further, characterized by a progressive decline in effective myelin debris clearance and the concurrent induction of neurotoxic reactive astrocytes [42]. Rather than definitively classifying this state as ‘exhaustion’—a concept that remains loosely defined in the context of spinal trauma—it is more accurately described as a dysfunctional, late-stage adaptation. The precise intracellular drivers of this chronic clearance failure in SCI remain incompletely elucidated. However, extrapolated evidence from neurodegenerative and brain injury models provides a robust mechanistic framework. Studies in these extraspinal paradigms suggest that such functional decline may stem from the downregulated or altered expression of key clearance receptors, including TREM2 [43] and CX3CR1 [44], or through the uncoupling of microglial motility from apoptotic cell recognition [45]. Furthermore, insights derived from brain pathology implicate disrupted lysosomal acidification [46] and impaired autophagic flux [47] as profound barriers to sustained debris degradation. Whether these specific receptor deficiencies and lysosomal/autophagic disruptions directly perpetuate chronic neuroinflammation and secondary neurodegeneration within the distinct microenvironment of the injured spinal cord constitutes a compelling theoretical framework that awaits definitive structural validation.

Microglial activation after SCI represents a continuous, multi-dimensional trajectory governed by transcriptional reprogramming, spatial compartments, and local microenvironmental cues. This includes (A) temporal evolution across standardized injury phases and (B) spatial-state mapping and evidence stratification. Under baseline conditions, homeostatic microglia (P2ry12^+^, Csf1r^+^) predominate. Post-injury, distinct functional subsets emerge within specific niches. Solid outlines denote states directly validated in primary murine SCI models via single-cell RNA sequencing [19,23], including transient non-lipid phagocytic (Lgals1^+^) subsets at the acute lesion core, proliferative (Mki67^+^) subsets at the scar border, and sustained DAM-like/lipid-processing (Lpl^+^, Trem2^+^, Ctsd^+^) subsets mediating debris clearance. Italicized text denotes conceptual states (e.g., chronic microglial ‘exhaustion’ or ‘senescence’) extrapolated from neurodegenerative paradigms [42,43,44,45,46,47]. While iron-handling transcriptomes (Fth1^+^) are confirmed in SCI [19], their precise contribution to functional exhaustion within the chronic lesion microenvironment awaits definitive in vivo validation.

### 3.3. In Vivo Imaging of Microglial Dynamics

Two-photon microscopy (TPM) has enabled real-time visualization of microglial behavior in vivo with high spatial and temporal resolution [48,49]. These studies reveal that microglia rapidly extend processes to injured axons, forming protective contacts that limit degeneration in the acute phase. Their targeted phagocytosis of damaged axonal fragments is essential for maintaining tissue homeostasis [50]. In the chronic phase, microglia remain activated but exhibit morphological simplification and reduced phagocytic dynamics. Although phagocytic structures persist, their functional relevance appears altered, suggesting a shift in microglial roles over time [51,52]. Microglia–neuron interactions are also dynamically regulated. Contact between microglia and neuronal somata increases shortly after injury and persists for weeks, indicating targeted surveillance. The normal inverse relationship between microglial activity and neuronal firing is transiently disrupted by injury, particularly in the presence of microhemorrhage, and gradually restored during recovery [53,54].

### 3.4. Dynamic Control of Microglial Function

The therapeutic efficacy of microglial depletion strategies in SCI is highly context-dependent, relying strictly on the precise timing of intervention, the specific pharmacological or genetic depletion model, and the duration of ablation.

Pre-injury and early acute depletion: Continuous depletion initiated prior to injury or immediately following trauma (e.g., via continuous dietary administration of CSF1R inhibitors like PLX5622/PLX3397 or conditional genetic ablation using Cx3cr1-DTR models) consistently exacerbates tissue damage and impairs functional recovery [38,55]. This detrimental outcome occurs because baseline microglia are essential for early wound compaction, rapid engulfment of necrotized tissue, and the assembly of a protective microglial border that physically confines peripheral leukocyte infiltration to the lesion core.

Delayed subacute depletion and repopulation: Conversely, temporal-specific microglial ablation initiated after the early protective scar border has stabilized yields striking therapeutic benefits. Direct evidence from a murine spinal cord contusion model demonstrates that delayed microglial depletion—achieved via dietary administration of the CSF1R inhibitor PLX5622 in male mice, specifically initiated at 7 days post-injury and maintained continuously for 4 weeks—significantly improves long-term neurological outcomes [38]. By sparing the critical acute window necessary for lesion containment, this delayed intervention selectively eliminates chronically activated microglial subpopulations that drive persistent secondary neurodegeneration [38] and maladaptive plasticity within autonomic circuitry [56]. Furthermore, upon cessation of the CSF1R inhibitor, the depleted parenchymal niche undergoes rapid repopulation solely derived from the proliferation of residual local microglial progenitors [57]. This dynamic cycle of delayed ablation and subsequent restorative repopulation profoundly attenuates chronic neuroinflammation, restricts secondary structural lesion expansion, and promotes sustained motor functional recovery [38]. Therefore, microglial depletion must not be viewed as a binary intervention, but rather as a highly stage-specific strategy that hinges on preserving early protective tissue encapsulation while precisely targeting chronic microglial toxicity.

Despite the epidemiological reality that SCI disproportionately affects males [1], a critical translational gap remains regarding sexual dimorphism within the microglial response and the IL-33/ST2 axis. Emerging broad neuroimmunology research demonstrates that male and female microglia exhibit fundamentally distinct transcriptomic baselines, colonization dynamics, and reactive profiles following CNS trauma, governed by both chromosomal complements and gonadal hormones. However, the vast majority of in vivo SCI studies evaluating microglial functional states—including the aforementioned delayed microglial depletion paradigms (conducted exclusively in male mice) [38] and rIL-33 administration protocols—have either disproportionately relied on male cohorts or failed to stratify molecular and behavioral outcomes by sex. Consequently, it remains unresolved whether the neuroprotective efficacy of IL-33 or the spatiotemporal transitions of DAM-like microglial subsets operate identically in female subjects. Overcoming this pervasive sex bias in preclinical study design is imperative to ensure that emerging immunomodulatory therapies are universally effective or appropriately tailored to sex-specific pathological trajectories.

## 4. Multidimensional Roles of IL-33 in SCI Repair

### 4.1. Biological Properties of IL-33 and Its Control of Microglial State Transitions

To establish a rigorous mechanistic framework and prevent invalid cross-disease extrapolation, the actions of IL-33/ST2 signaling in microglial regulation are herein evaluated across three explicit tiers of evidence: direct evidence derived from traumatic SCI models; supporting evidence drawn from related acute central nervous system (CNS) injuries (such as traumatic brain injury and ischemic stroke); and hypotheses awaiting SCI validation, inferred from neurodegenerative or peripheral immune contexts.

Interleukin-33 (IL-33), a member of the IL-1 family, functions as an alarmin that is rapidly released following SCI and contributes to neural repair. In the CNS, IL-33 is primarily derived from oligodendrocytes and astrocytes [4]. Its effects on microglia are highly dependent on both timing and context.

Upon mechanical insult to the spinal cord, necrotized glial cells rapidly discharge nuclear-sequestered IL-33 into the extracellular matrix. In vivo observations in rodent contusion models verify that this endogenous alarmin recruits myeloid populations to the injury site and attenuates the acute surge of pro-inflammatory cytokines (such as TNF-α and IL-1β), thereby substantially reducing secondary lesion volume [58]. However, the highly granular cellular mechanisms often attributed to IL-33 in this context—such as instructing targeted microglial chemotaxis, amplifying myelin phagocytosis, and orchestrating structural border assembly—are derived primarily from ischemic stroke pathology, where the ST2 receptor regulates monocyte-derived macrophages [6]. While highly plausible in spinal trauma, the exact temporal and spatial orchestration of microglial migration and boundary formation by IL-33 requires targeted experimental confirmation to bridge the gap between cerebral and spinal lesions.

Beyond acute immunomodulation, exploring the downstream intracellular cascades and multicellular crosstalk of IL-33 requires careful stratification of evidence, as distinct CNS microenvironments are frequently conflated. For instance, in a rodent model of epilepsy [59], IL-33 has been shown to limit neuronal apoptosis through the coordinated regulation of Bcl-2 family proteins and attenuation of endoplasmic reticulum stress. Independent of this, mechanistic insights from cerebral injury models demonstrate that IL-33 expands regulatory T (Treg) cell populations via ST2 activation, enabling Treg-derived amphiregulin (AREG) to restrain reactive astrogliosis [60]. It is imperative to clarify that these anti-apoptotic intracellular cascades [59] and the IL-33–Treg–AREG intercellular axis [60] represent isolated findings from disparate disease states. They have neither been serially linked nor formally tested within the post-traumatic spinal cord. Consequently, whether these parallel pathways operate concurrently to modulate glial scar rigidity and support functional circuit repair in SCI remains to be verified.

Microglia express the canonical IL-33 receptor ST2, through which the alarmin modulates phagocytic capacity and synaptic pruning during neurodevelopmental remodeling [61,62]. Whether this pathway is reactivated following adult spinal trauma to instruct targeted circuit reorganization—or conversely, to drive aberrant synapse elimination—remains to be empirically determined. Furthermore, the specific transcriptional targets of IL-33 in microglia require rigorous contextualization. High-resolution transcriptomic profiling in Alzheimer’s disease (AD) models [63] has demonstrated that IL-33 reprograms microglia to upregulate extracellular matrix-modifying enzymes (such as *Adamts4* and *Mmp14*) alongside phagocytosis-associated genes (including *Marco* and *Gas7*). While fundamental biology dictates that these enzymes and receptors degrade inhibitory chondroitin sulfate proteoglycans (CSPGs) [64] or facilitate actin-driven membrane dynamics [65,66], respectively, their IL-33-driven upregulation has only been established in the amyloid-burdened brain. Whether exogenous IL-33 initiates an analogous transcriptional program to clear myelin debris and actively degrade the glial scar in the traumatically injured spinal cord represents a putative mechanism that has yet to be corroborated in neurotrauma models.

Beyond transcriptional regulation, metabolic adaptation represents another pivotal dimension of IL-33 action. In neurodevelopmental contexts [67,68], this alarmin signals through the PI3K–AKT axis to elevate both mitochondrial respiration and glycolysis, thereby meeting the heightened bioenergetic demand required for microglial phagocytosis. Pharmacological blockade of these metabolic pathways abolishes phagocytic functional adaptation during physiological remodeling. Although these developmental findings suggest that the IL-33/ST2 axis integrates metabolic and transcriptional programs, whether a parallel bioenergetic reprogramming operates in spinal microglia following traumatic injury remains a critical knowledge gap that necessitates rigorous metabolic profiling in future spinal contusion studies.

### 4.2. Temporal Dynamics of IL-33 and Its Evolving Microglial Regulation Post-SCI

Following primary mechanical trauma, endogenous IL-33 functions as a rapid-response alarmin. As noted above, damaged spinal astrocytes and oligodendrocytes swiftly release nuclear-stored IL-33. Primary studies of spinal contusion and crush injuries confirm that this early peak, occurring within 24 h, is vital for blunting the acute neuroinflammatory response [6,58]. While this transient surge is protective, the sustained elevation of IL-33 signaling observed in chronic stages—likely maintained by ongoing vascular and astroglial remodeling—conversely drives fibrotic scar stabilization [6].

However, the precise downstream intracellular networks governing IL-33-mediated microglial regulation remain largely deduced from non-traumatic conditions. For instance, high-resolution studies in AD models reveal that acute IL-33 engages microglial ST2 receptors to activate NF-κB and AKT pathways, which subsequently upregulate the master transcription factor PU.1. This IL-33–PU.1 axis drives a sophisticated transcriptional program encompassing repair-associated genes (e.g., *Arg1* and *Mrc1*/*CD206*) and anti-inflammatory cytokines (including IL-10 and TGF-β) [63]. While this mechanistic framework elegantly illustrates how IL-33 might bypass simplistic binary polarization to foster a protective tissue microenvironment, its direct execution within the unique pathology of SCI serves as a theoretical model that must be strictly tested against the specialized cellular architecture of the injured spinal cord.

**Figure 3:**
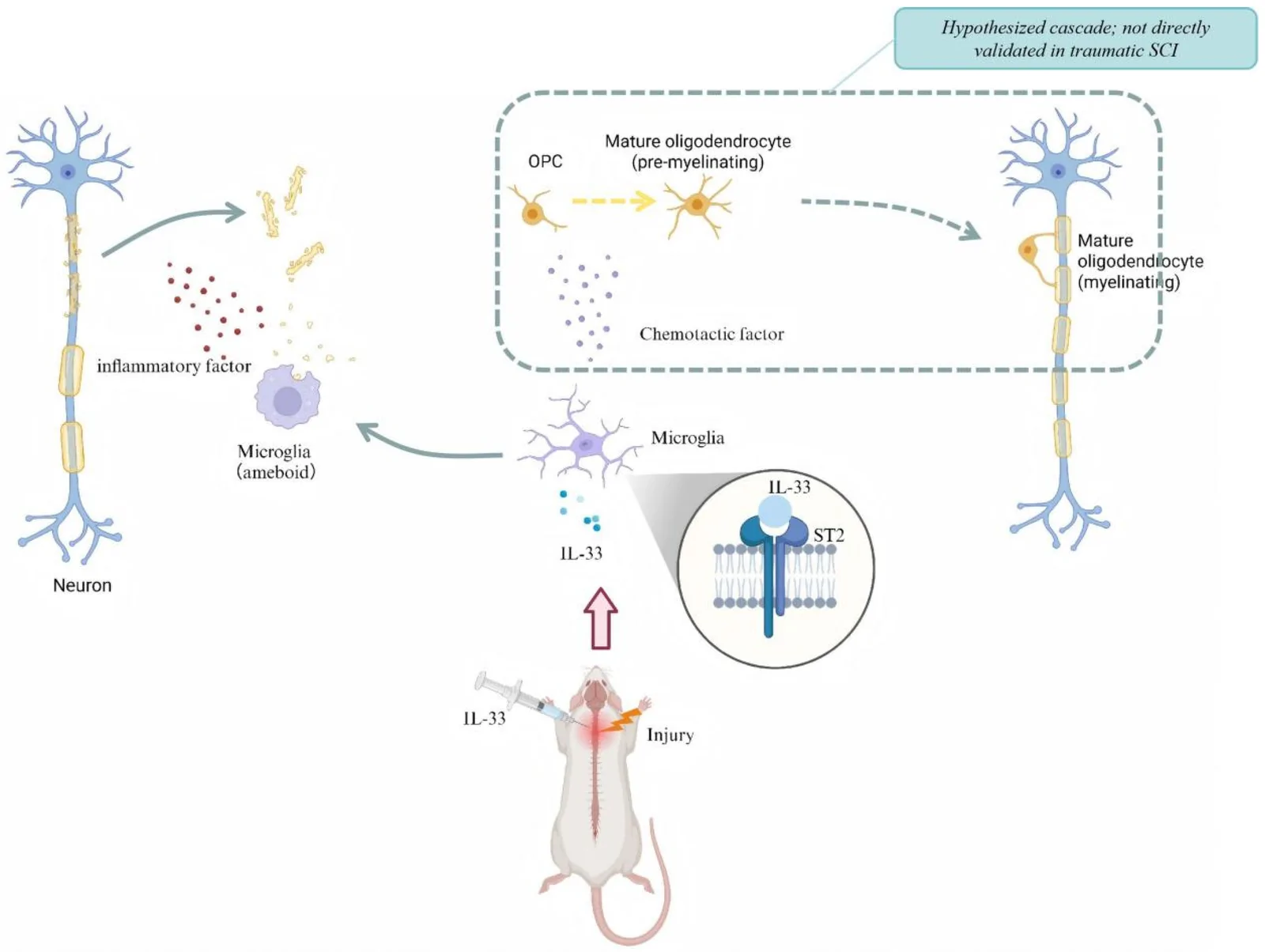
Proposed working model of IL-33-mediated microglial regulation and hypothesized neuroglial crosstalk following SCI.

Hypotheses Awaiting SCI Validation: Beyond acute immunomodulation, several downstream functional roles attributed to IL-33 represent candidate pathways currently lacking direct confirmation in spinal trauma. For instance, insights from AD demonstrate that IL-33 induces a sequential transition from chemotactic to phagocytic microglial states via ST2 signaling and VCAM1–ApoE interactions, enabling efficient clearance of pathological substrates [69]. By analogy, IL-33 is hypothesized to mobilize spinal microglia toward inhibitory myelin debris to accelerate its clearance, thereby removing a major physical barrier to axonal regeneration. Furthermore, proposed cytoprotective functions—such as preserving microglial viability by limiting excessive autophagy and suppressing cellular stress responses via NF-κB and AP-1 signaling [70,71]—remain speculative concepts that await formal validation in SCI models (Figure 3).

Collectively, IL-33 orchestrates microglial responses through coordinated regulation of inflammation, metabolism, survival, and phagocytosis. These integrated effects position the IL-33/ST2 axis as a central regulator of neuroinflammation and a promising therapeutic target for SCI repair.

Local mechanical trauma or exogenous delivery of IL-33 at the spinal lesion site engages microglial ST2 receptors. Solid arrows depict processes with direct in vivo support in acute SCI models, specifically driving acute microglial activation and the initial steps of phagocytic clearance of inhibitory myelin debris. In parallel, dashed arrows represent a highly speculative downstream cascade. It is hypothesized—largely by extrapolating from primary demyelinating models (e.g., cuprizone or EAE)—that efficient debris clearance and subsequent IL-33-stimulated microglial secretion of specific trophic factors might establish a permissive microenvironment for the recruitment and differentiation of oligodendrocyte precursor cells (OPCs) into mature oligodendrocytes. It must be emphasized that this sequential pathway culminating in structural remyelination remains theoretically proposed and currently lacks direct empirical validation within the complex, non-permissive pathology of traumatic SCI.

## 5. Therapeutic Strategies Targeting IL-33 and Microglia

IL-33 exhibits robust neuroprotective effects in experimental models of SCI. However, its clinical translation remains limited by challenges in achieving efficient delivery of recombinant proteins to the lesion site and in developing safe and controllable gene-based interventions.

Direct intervention using recombinant IL-33 (rIL-33) has demonstrated substantial neuroprotective potential. Specifically, in a murine spinal cord contusion model, systemic intraperitoneal administration of rIL-33 (typically initiated immediately post-injury and sustained through the acute phase) has been shown to modulate receptor dynamics by reducing IL-1RAcP expression while upregulating ST2 [72]. This systemic regimen effectively attenuates gross tissue loss, limits demyelination, and reduces reactive astrogliosis, concurrently promoting a sustained reparative microglia/macrophage phenotype and suppressing both T cell infiltration and TNF-α expression [72]. However, while these endpoint functional and histological benefits are firmly established by this specific experimental design, translating them into a generalized mechanistic protocol requires extreme caution. Crucial parameters—including the precise dose–response relationship, the strict boundaries of the optimal therapeutic time window, and whether exogenous rIL-33 explicitly drives physical microglial border compaction—were not unequivocally resolved by these studies and remain vital questions awaiting dedicated in vivo investigation.

Beyond direct traumatic SCI repair, targeting the IL-33/ST2 axis offers valuable translational insights for non-traumatic spinal neuropathies. Specifically, lentiviral delivery of short hairpin RNA targeting IL-33 (LV-shIL-33) effectively silences local spinal IL-33 expression, thereby dampening neuroinflammatory cascades and alleviating neuropathic pain in a rodent model of radicular pain associated with intervertebral disc herniation [73].

While this gene-silencing strategy underscores the potential of targeted IL-33 inhibition in suppressing chronic neuroinflammatory pain, it originates from a non-traumatic radicular neuropathy model rather than a primary traumatic spinal contusion or transection model. Consequently, this anti-nociceptive approach must not be conflated with acute SCI tissue repair strategies, where early IL-33 signaling is indispensably required for microglial encapsulation and debris clearance. Whether delayed, region-specific IL-33 knockdown can selectively mitigate post-traumatic neuropathic pain without compromising structural tissue integrity remains a critical hypothesis awaiting dedicated empirical testing in SCI paradigms.

Modulation of IL-33 signaling can also be achieved through receptor-based interventions. Soluble ST2 (sST2) functions as a decoy receptor that sequesters IL-33, thereby preventing its interaction with membrane-bound ST2. This approach attenuates inflammatory signaling and may offer therapeutic benefit in conditions characterized by excessive immune activation [74]. Similarly, antisense oligonucleotides targeting IL-33R/ST2 mRNA reduce receptor expression and alleviate IL-33-induced hyperalgesia, supporting the feasibility of targeting this pathway for pain modulation [75,76].

Beyond local neuroprotective efficacy, the systemic safety profile of IL-33 modulation warrants stringent evaluation prior to clinical translation. Evidence from ischemic stroke models indicates that while central IL-33 administration dampens local neuroinflammation, it can inadvertently provoke peripheral immune dysfunction, culminating in systemic immunosuppression and heightened susceptibility to secondary bacterial infections [77].

Rather than precluding IL-33 therapy, these findings serve as a critical translational caution. To circumvent the inherent risks of post-injury systemic immunosuppression, we propose that future SCI-targeted IL-33 therapeutic paradigms must pivot toward lesion-site-restricted delivery platforms. Advanced biomaterial strategies—such as injectable hydrogels, which have independently proven highly effective in enhancing extracellular matrix remodeling and tissue repair following SCI [78], or targeted nanocarriers—offer promising theoretical avenues. By physically restricting IL-33 release strictly to the compromised spinal parenchyma, these engineered platforms could conceptually minimize off-target systemic spillover. It is imperative to emphasize, however, that tailored localized IL-33 delivery systems for traumatic SCI currently lack direct empirical validation and represent a critical frontier for future preclinical development.

To systematically evaluate the translational potential of these diverse therapeutic approaches, it is essential to rigorously distinguish between interventions validated directly within primary traumatic SCI and those extrapolated from related neuropathies or brain injuries. A comprehensive classification of current IL-33/ST2-targeted strategies, explicitly stratified by their underlying evidence tiers and translational caveats, is summarized in Table 3.

## 6. Limitations of the Review

While this narrative synthesis provides a structured overview of microglial plasticity and the IL-33/ST2 axis, several methodological limitations must be acknowledged. Primarily, our literature selection criteria strictly excluded non-English publications and non-peer-reviewed preprints. While this ensured a baseline of peer-reviewed quality, it may have inadvertently omitted pertinent early-stage mechanistic discoveries or relevant international findings. Furthermore, this review is inherently susceptible to publication bias; studies demonstrating robust neuroprotective effects or positive immunomodulatory outcomes of IL-33 are statistically more likely to be published than those yielding null or negative results. Finally, as emphasized throughout our tiered evidence framework, the deliberate integration of extrapolated data from non-SCI models means that a substantial portion of the discussed downstream molecular cascades remains theoretical in the context of primary spinal trauma, highlighting the necessity for continued empirical validation.

## 7. Conclusions

In summary, traumatic SCI triggers a complex, dynamic neuroinflammatory cascade in which microenvironmental imbalance remains a primary obstacle to repair. Microglia function as central regulators of this process, with their functional states evolving over time—potentially shifting from early protective responses toward chronic or dysregulated phenotypes. This functional plasticity underscores the need for precise, context-dependent control rather than broad microglial suppression.

The IL-33/ST2 axis represents a candidate pathway with emerging preclinical support that links alarmin signaling with microglial functional reprogramming. Current evidence suggests that IL-33 modulates key aspects of microglial physiology, including phagocytic debris clearance, bioenergetic adaptation, and tissue-restricting border formation. However, important evidence gaps remain: much of the mechanistic framework is derived from non-traumatic or indirect disease models, the precise intracellular networks dictating microglial state transitions downstream of ST2 are not yet fully elucidated, and direct validation in human SCI tissue is severely limited. Moreover, microglial responses to IL-33 appear tightly constrained by spatial and temporal context, reflecting a complex biological balance that warrants cautious interpretation.

Moving from preclinical observations to a definitive understanding of the IL-33–microglia axis will require addressing critical unresolved issues. Rather than assuming broad therapeutic efficacy, future studies must systematically evaluate four key testable domains:

1. Incorporating Aged Injury Models: As regenerative capacity declines with age, preclinical models must pivot from relying exclusively on young adult rodents. Evaluating the IL-33/ST2 axis in aged models is essential to determine whether microglial senescence alters reparative responses and whether immunomodulation remains efficacious in older populations.
2. Establishing Human-Translational Biomarkers: Given the severe limitations of direct validation in human SCI tissue, establishing accessible proxy metrics is critical. Future clinical profiling should prioritize the longitudinal quantification of IL-33 and soluble ST2 (sST2) in human cerebrospinal fluid (CSF) and serum to map these biomarker trajectories against injury severity, neuroinflammation, and clinical recovery.
3. Defining the Optimal Therapeutic Window and Cell-Specific Actions: Experimental designs must tightly control dosing windows and leverage high-resolution genetic tools (e.g., Cx3cr1-CreER lineage tracing) to determine when exogenous IL-33 facilitates border compaction without triggering secondary damage.
4. Evaluating Long-Term Safety and Systemic Immune Risks: Preclinical trials must incorporate rigorous systemic immune metrics to ensure that localized IL-33 modulation does not induce peripheral immunosuppression or heighten vulnerability to post-injury complications, such as pneumonia.

Addressing these highly specific scientific questions will be essential to establish whether targeting microglial functional plasticity via the IL-33/ST2 axis can ultimately be translated from a promising preclinical concept into a controllable, safe, and effective therapeutic strategy for spinal cord repair.

## Figures and Tables

**Table 1:** Temporal definitions of SCI phases across experimental models and clinical guidelines.

Injury Phase	Preclinical Murine Models (Adopted in This Review)	Clinical (Human) Consensus Guidelines	Primary Pathological Hallmarks in Murine Models
Acute	0–3 days	0–48 h	Microhemorrhage, ischemia, initial ROS burst, massive cell death, rapid microglial activation (Lgals1^+^, CD68^+^), and neutrophil infiltration.
Subacute	3–28 days (4 weeks)	2 days–2 weeks	Microglial transition to DAM-like/lipid-processing states (Trem2^+^), early scar formation, adaptive immune cell (T/B cell) recruitment, and debris phagocytosis.
Chronic	>28 days	>6 months	Stabilization of fibrotic/astrocytic scar, microglial exhaustion (iron-handling subsets), aborted axonal regeneration, and persistent low-grade neuroinflammation.

**Table 2:** Summary of key microglial subpopulations across CNS pathologies graded by evidence tiers in traumatic SCI.

Microglial Subpopulation	Defining Marker Genes	Evidence Tier & Source Model	Validation Status & Dynamics in Traumatic SCI	Ref.
Homeostatic Microglia	*P2ry12*, *Csf1r*, *Cx3cr1*, *Tmem119*, *Hexb*	Tier 1: Direct Traumatic SCI	Confirmed. Rapidly downregulated post-injury in the lesion core; gradually restored in spared parenchyma during chronic recovery.	[18,19,22]
DAM-like/Lipid-processing Microglia	*Trem2*, *Apoe*, *Lpl*, *Ctsd*, *Axl*, *Spp1*	Tier 1: Direct Traumatic SCI (Originally Tier 3 in AD models)	Confirmed. Prominent in the subacute and chronic lesion core. Reprogrammed to mediate myelin debris engulfment, lipid breakdown, and border formation.	[19,23,32]
Proliferative/Cycling Microglia	*Mki67*, *Top2a*, *Cdk1*, *Prc1*	Tier 1: Direct Traumatic SCI	Confirmed. Rapid local self-renewal to fill depleted niches; peaks strictly within 3–7 days post-injury at the peri-lesion boundary.	[18,19]
Non-lipid Phagocytic/Inflammatory Microglia	*Lgals1*, *Cd68*, *Clec7a*, *Csf1*	Tier 1: Direct Traumatic SCI	Confirmed. Mediates acute phagocytosis of cellular debris and secretes pro-inflammatory cytokines; largely cleared by the chronic stage.	[19]
Antigen-presenting Microglia	*H2-Aa*, *H2-Ab1*, *Cd74*, *Ciita*	Tier 1: Direct Traumatic SCI	Confirmed. Expands during the subacute-to-chronic transition, facilitating localized MHC-II antigen presentation to adaptive immune cells.	[19,22,23]
Repair-associated Microglia (RAM)	*Vegfa* (*VEGF-A secretion*)	Tier 2: Related CNS Injury (Cerebrovascular injury model)	Extrapolated/Pending. Putative role in supporting BSCB integrity in early SCI; lacks direct validation separating RAMs from general phagocytic subsets in SCI.	[31]
Interferon-responsive Microglia (IRM)	*Isg15*, *Irf7*, *Stat1*, *Ifit1*	Tier 2: Related CNS Injury (Viral/EAE models)	Minor Subset. Identified via scRNA-seq as a minor, transient subset in acute SCI; specific functional relevance in trauma remains largely unknown.	[19,22]
CD11c^+^/OPN-producing Microglia	*Itgax* (CD11c), *Spp1* (OPN)	Tier 3: Neurodevelopment & AD Hypothesis	Pending. Well-defined in development and AD, but evidence for a distinct, stable CD11c^+^/OPN-dependent subset purely in primary traumatic SCI is lacking.	[28]
Dark Microglia	Ultrastructural stress features, *Trem2*-independent	Tier 3: Neurodegeneration (AD/Aging) Hypothesis	Unclear. Heavily linked to toxic lipid secretion and synaptic stress in neurodegeneration; their presence and relevance in acute/chronic SCI are unverified.	[29,30]

**Table 3:** Classification of IL-33/ST2-targeted strategies: direct evidence in traumatic SCI versus indirect insights from non-SCI models.

Therapeutic Strategy/Intervention	Disease/Model	Mechanistic Target & Functional Outcome	Evidence Tier	Relevance & Translational Caveat for Traumatic SCI	Ref.
Group 1: Direct Evidence in Traumatic SCI Paradigms					
Recombinant IL-33 (rIL-33) (*Acute local*/*systemic administration*)	Rodent spinal contusion/compression model	Activates microglial ST2; enhances early reparative phenotypes; dampens acute TNF-α/IL-1β surge; attenuates demyelination.	Level 1 (Direct SCI)	Moderate (Pending Replication): While it establishes exogenous IL-33 as a potential acute immunomodulator, current evidence stems from a solitary, unreplicated study. Crucial parameters—including exact optimal dosing, the precise therapeutic window, and local delivery requirements—remain ambiguous and require rigorous independent validation before claiming high translational readiness.	[72]
ST2/IL-33 Genetic Knockout	Mouse spinal cord contusion model	Loss of IL-33/ST2 signaling impairs acute myeloid cell recruitment, expands secondary lesion volume, and exacerbates motor deficits.	Level 1 (Direct SCI)	High (Endogenous role): Confirms the indispensable protective role of early endogenous IL-33/ST2 activation in primary spinal trauma.	[58]
Group 2: Indirect Evidence & Translational Insights from Non-SCI Paradigms					
LV-shIL-33 (*Lentiviral IL-33 knockdown*)	Intervertebral disc herniation (*Radicular pain model*)	Silences spinal IL-33 expression; attenuates local microglial overactivation and neuroinflammatory pain signaling.	Level 2 (Indirect Pain Model)	Moderate (Pain-specific): Relevant for post-injury chronic neuropathic pain management. However, acute knockdown in primary SCI is strictly contraindicated as it disrupts early protective containment.	[73]
Systemic rIL-33 Administration	Focal ischemic stroke/Cortical TBI models	Accelerates microglial state transitions; however, inadvertently provokes systemic immunosuppression and high secondary infection risk.	Level 2 (Indirect Brain Injury)	Moderate (Safety Caution): Strongly warns against non-targeted systemic IL-33 delivery in CNS trauma, underscoring the necessity for lesion-site-restricted platforms (e.g., hydrogels) in future SCI trials.	[77]
IL-33 Modulation of A-type K^+^ Channels	Nociceptive pain/DRG hyperexcitability	Exogenous IL-33 directly inhibits A-type K^+^ channels in DRG sensory neurons, inducing sensory hyperexcitability and hyperalgesia.	Level 3 (Indirect Peripheral Pain)	Low (Peripheral Neuromodulation): Demonstrates IL-33′s capacity to directly drive peripheral sensory pain. Not applicable to central microglial debris clearance or primary structural tissue repair in SCI.	[74]
Nociceptor ST2 Signaling Blockade	Vibration-induced muscle pain (*HAVS model*)	Modulates ST2 signaling strictly within peripheral nociceptors to drive mechanical hyperalgesia following vibration injury.	Level 3 (Indirect Muscle Pain)	Low (Mechanistic analogy): Highly specific to peripheral nociceptor sensitization following physical vibration; serves solely as a reference for pain processing, without relevance to primary SCI repair mechanisms.	[75]

## Data Availability

No new data were created or analyzed in this study. Data sharing is not applicable to this article.
